# High Resolution Genotyping of Clinical *Aspergillus flavus* Isolates from India Using Microsatellites

**DOI:** 10.1371/journal.pone.0016086

**Published:** 2011-01-17

**Authors:** Shivaprakash M. Rudramurthy, Hanneke A. de Valk, Arunaloke Chakrabarti, Jacques F. G. M. Meis, Corné H. W. Klaassen

**Affiliations:** 1 Mycology Division, Department of Medical Microbiology, Post Graduate Institute of Medical Education and Research, Chandigarh, India; 2 Department of Medical Microbiology and Infectious Diseases, Canisius Wilhelmina Hospital, Nijmegen, The Netherlands; Duke University Medical Center, United States of America

## Abstract

**Background:**

Worldwide, *Aspergillus flavus* is the second leading cause of allergic, invasive and colonizing fungal diseases in humans. However, it is the most common species causing fungal rhinosinusitis and eye infections in tropical countries. Despite the growing challenges due to *A. flavus*, the molecular epidemiology of this fungus has not been well studied. We evaluated the use of microsatellites for high resolution genotyping of *A. flavus* from India and a possible connection between clinical presentation and genotype of the involved isolate.

**Methodology/Principal Findings:**

A panel of nine microsatellite markers were selected from the genome of *A. flavus* NRRL 3357. These markers were used to type 162 clinical isolates of *A. flavus*. All nine markers proved to be polymorphic displaying up to 33 alleles per marker. Thirteen isolates proved to be a mixture of different genotypes. Among the 149 pure isolates, 124 different genotypes could be recognized. The discriminatory power (D) for the individual markers ranged from 0.657 to 0.954. The D value of the panel of nine markers combined was 0.997. The multiplex multicolor approach was instrumental in rapid typing of a large number of isolates. There was no correlation between genotype and the clinical presentation of the infection.

**Conclusions/Significance:**

There is a large genotypic diversity in clinical *A. flavus* isolates from India. The presence of more than one genotype in clinical samples illustrates the possibility that persons may be colonized by multiple genotypes and that any isolate from a clinical specimen is not necessarily the one actually causing infection. Microsatellites are excellent typing targets for discriminating between *A. flavus* isolates from various origins.

## Introduction

*Aspergillus* species are known to cause a wide range of disorders including allergic, colonizing and invasive disease in immunocompromised as well as immunocompetent hosts [1]. *A. fumigatus* is the predominant etiological agent in patients with aspergillosis followed by *A. flavus* [1]–[4]. However, in certain countries such as India, Saudi Arabia and Sudan, *A. flavus* is the predominant etiological agent in patients with fungal rhinosinusitis and endophthalmitis [4]–[6]. A. *flavus* has been reported to cause outbreaks of mucocutaneous and subcutaneous aspergillosis [4], [5], [7], [8]. In immunosuppressed mice it was observed that much lower inocula of *A. flavus* spores could kill animals compared to *A. fumigatus* spores [9]–[11]. The agent is also known to cause environmental aflatoxin contamination in crops like maize, cottonseed, almond, pistachio and peanuts, which leads to substantial economic damage worldwide [12].

Despite the growing challenge due to *A. flavus*, the molecular epidemiology of this fungus has not been well studied. Molecular typing of *A. flavus* has been hindered by lack of discriminatory and exchangeable techniques for this fungus. Several genotypic methods have been utilized for the molecular typing of this fungus which includes random amplified polymorphic DNA (RAPD) [13], [14] restriction fragment length polymorphism (RFLP) [15], [16] and amplified fragment length polymorphism (AFLP) [17]. These techniques all utilize complex banding patterns to discriminate between isolates. These techniques often have a poor inter-laboratory reproducibility and the exchange of results between laboratories is difficult. In this context, molecular typing using microsatellites yields multiple advantages such as a high discriminatory power, high reproducibility and easy exchange of results [18]. Microsatellite based typing has been well established for *A. fumigatus* [19]–[26]. The technique is based on PCR amplification of short tandemly repeated DNA motifs of 2–10 bp that are abundantly present in the genomes of fungi followed by the determination of fragment size by capillary electrophoresis (CE). Sizing by CE has a resolution below one nucleotide which is essential for the high level of reproducibility of the technique [19]–[22], [24], [26]. The number of repeats is extrapolated from the fragment size. We developed a multiplex, multicolor microsatellite panel for genotyping of *A. flavus* and investigated whether there might be a link between *A. flavus* genotype and clinical presentation of the infection. This work was presented at the 4th ‘Advances Against Aspergillosis’ (AAA), Rome, Italy 4–6 February 2010.

## Materials and Methods

### Ethics statement

This study is approved by the Institutional Ethics Committee of the Postgraduate Institute of Medical Education and Research, Chandigarh, India. No informed consent was obtained. The ethics committee allowed the use of clinical isolates without informed consent provided that no individual patient would be identified. All data were analyzed anonymously.

### Isolates

A collection of 162 clinical isolates of *A. flavus* isolated from the patients attending the Postgraduate Institute of Medical Education and Research, Chandigarh, India and stored at the National Culture Collection of Pathogenic Fungi (NCCPF), PGIMER, Chandigarh, India was included in the study. The isolates were recovered from clinical samples collected over a two year period (January 2007 through December 2008). PGIMER, Chandigarh is a tertiary care referral medical centre catering the need of patients from provinces (states) of north India including Punjab, Haryana, Himachal Pradesh, western Uttar Pradesh, and northern Rajasthan (covering an area over 350,000 sq km.). The strains were isolated from patients with allergic fungal rhinosinusitis (AFRS, n = 75), invasive fungal rhinosinusitis (n = 23), pulmonary aspergillosis (n = 26), keratitis (n = 23), endophthalmitis (n = 5), or others (n = 10). All isolates were from different patients. *A. flavus* NRRL 3357 was obtained from the Centraalbureau voor Schimmelcultures, Utrecht, The Netherlands and was used as the control strain in all experiments. The identity of the isolates were verified by taxonomic criteria [27] and further confirmed by amplified fragment length polymorphism (AFLP) analysis [22].

### DNA isolation

For the isolation of DNA a pre-wetted cotton swab was saturated with conidia from a freshly grown sporulating culture. Conidia were resuspended in a vial containing 350 µl lysis buffer and MagNA Lyser Green Beads (Roche Diagnostics, Almere, the Netherlands) and subjected to mechanical lyses in a MagNA Lyser instrument (Roche Diagnostics) for 30s at 6500 rpm. Next, the DNA was extracted and purified using MagNA Pure DNA isolation kit III according to the recommendations of the manufacturer (Roche Diagnostics) on a MagNA Pure LC instrument. The yield and purity of the DNA were estimated by UV absorbance measurements.

### Microsatellite typing

Candidate microsatellite markers were identified in the genomic sequences of *A. flavus* NRRL 3357 (Genbank entry AAIH00000000) using the Tandem Repeats Finder software [28]. A microsatellite panel of nine markers consisting of three dinucleotide repeat markers, three trinucleotide repeat markers and three tetranucleotide repeat markers was selected from the candidate markers following previously described criteria [21]. PCR amplification primers for each of the markers were designed using Primer3 software (Table 1). Three sets (AflaSTR2, AflaSTR3 and AflaSTR4 respectively) of 3 markers each (sub numbered A, B and C, respectively) were amplified using a multicolor multiplex PCR approach. Within each set, one of the amplification primers carried a fluorescent label consisting of either FAM (6-carboxyfluorescein), HEX (hexachlorofluorescein) or TAMRA (tetramethylrhodamine) (Table 1). Each 25 µl amplification reaction contained 0.5 µM of each amplification primer, approximately 1 ng of genomic DNA, 1 U FastStart Taq DNA polymerase (Roche diagnostics), 2 mM MgCl_2_, and 0.2 mM dNTP's in 1× reaction buffer (Roche diagnostics). The amplification profile consisted of a 10 min denaturation/activation step at 94°C followed by 35 cycles of 94°C for 30 s, 60°C for 30 s and 72°C for 1 min. After an additional 10 min incubation at 72°C, the reactions were cooled to room temperature.

**Table 1:** Basic characteristics of the selected microsatellite markers.

Marker	Repeat unit	Labeled primer sequence (5′-3′)	Unlabeled primer sequence (5′-3′)^1^	No. alleles (range)
AflaSTR2A	CA	FAM-TCTCTCTGGGGTGAAGTCTGA	G TCTGCCTGTACGCCTCTCTT	33 (10–75)
AflaSTR2B	GA	HEX-GGTTCTCGAGTCGGTTTGAT	GAGACCTTTTGCAATCAGCA	7 (7–17)
AflaSTR2C	CA	TAMRA-AATCAAGAGCAAGACGTCCA	G TTGAGGCGCTTTCCAACTAC	22 (10–38)
AflaSTR3A	AAG	FAM-CATTGCATGTTAGCCCAAAG	GGTAATCCAGATGCGCTGTT	13 (7–28)
AflaSTR3B	AAG	HEX-CCTCGATGGTGAGAGGCTTA	GGGATGTATTTCGAGGTCCTT	30 (8–40)
AflaSTR3C	AAT	TAMRA-CCAAACATGGCAGAATCAAA	GTTGAGACGGAGAAGCGAAG	24 (5–35)
AflaSTR4A	TCTT	FAM-TCATCAAGATACAACACCCAGCTA	G AGGTGTTTGGGTGTCCTTGT	6 (3–10)
AflaSTR4B	TAGG	HEX-CTGAAAGGGTAAGGGGAAGG	GCAGGGAATACAGCACAACG	9 (5–16)
AflaSTR4C	AAAG	TAMRA-CATGAAAAGTATGGCGCAAA	GATGGTTTCTCGCGATTTGT	9 (5–17)

1 The underlined residues are a mismatch to the genomic sequence of *A. flavus* NRRL 3357. These were introduced to minimize the formation of minus-A peaks, a well known PCR artefact that may complicate interpretation of the results [23].

### AFLP analysis

Approximately 50 ng of genomic DNA was subjected to a combined restriction-ligation procedure containing 50 pmol of HpyCH4 IV adapter, 50 pmol Mse I adapter, 2 U of HpyCH4 IV (New England Biolabs, Beverly, MA, USA), 2 U of Mse I (New England Biolabs) and 1 U of T4 DNA ligase (Promega, Leiden, The Netherlands) in a total volume of 20 µl of 1× reaction buffer for 1 hour at 20°C. Adapters were made by mixing equimolar amounts of complementary oligonucleotides (5′-CTCGTAGACTGCGTACC-3′ and 5′-CGGGTACGCAGTC -3′ for HpyCH4 IV; 5′-GACGATGAGTCCTGAC-3′ and 5′- TAGTCAGGACTCAT –3′ for Mse I) heated to 95°C and subsequently slowly cooled to ambient temperature. Next, the mixture was diluted five times with 10 mM Tris/HCl pH 8.3. One microliter of the diluted restriction-ligation mixture was used for amplification in a volume of 25 µl under the following conditions: 1 µM HpyCH4 IV primer with one selective residue (5′-Flu-GTAGACTGCGTACCCGTC-3′), 1 µM MseI primer with four selective residues (5′-GATGAGTCCTGACTAATGAA-3′), 0.2 mM of each dNTP and 1 U of Taq DNA polymerase (Roche Diagnostics) in 1× reaction buffer containing 1.5 mM MgCl_2_.

Amplification was done as follows. After an initial denaturation step at 94°C for 4 min in the first 20 cycles a touchdown procedure was applied: 15 s denaturation at 94°C; 15 s annealing at 66°C with the temperature for each successive cycle lowered by 0.5°C and 1 min of extension at 72°C. Cycling was then continued for further 30 cycles with an annealing temperature of 56°C. After completion of the cycles, an incubation at 72°C for 10 min was included before the reactions were cooled to room temperature.

### Capillary electrophoresis

Reaction products were diluted 10 fold with distilled water. One microliter of diluted products was combined with 0.25 µl of ET400-R size marker (GE Healthcare, Diegem, Belgium) and 8.75 µl of distilled water. Samples were denatured for 1 min at 94°C and quickly cooled to 4°C before injecting onto a MegaBACE 500 automated DNA analysis platform equipped with a 48 capillary array as recommended by the manufacturer (GE Healthcare). Microsatellite data was analyzed using Fragment Profiler 1.2 software (GE Healthcare). Assignment of repeat numbers was relative to the results obtained using *A. flavus* NRRL 3357. In concordance with the analysis by the Tandem Repeats Finder software, the genotype of the NRRL 3357 strain was 16-16-19-19-17-15-11-12-11 for markers 2A-2B-2C-3A-3B-3C-4A-4B- and 4C, respectively.

### Data analysis

Typing data was imported into BioNumerics v5.1 software (Applied Maths, Sint-Martens-Latem, Belgium). Microsatellite data was analyzed using the multistate categorical similarity coefficient. AFLP data was analyzed by UPGMA clustering using the Pearson correlation coefficient and was restricted to fragments in the range of 50–300 nucleotides.

### Discriminatory power

The discriminatory power of the microsatellite markers was calculated using Simpson's index of diversity (D). This index is a statistical measure that any two randomly chosen isolates are of the same genotype for a given (combination of) marker(s). A ‘D’ value of 1 indicates all isolates to be different whereas a ‘D’ value of 0 indicates all isolates to be identical.

## Results

A panel of nine microsatellite markers [including three sets of three dinucleotide repeat loci (2A +2B+2C), three trinucleotide repeat loci (3A+3B+3C) and three tetranucleotide repeat loci (4A+4B+4C)] were selected from the genome of *A. flavus*, as per the inclusion and exclusion criteria described for the STRA*f* typing assay for *A. fumigatus* [21]. All nine markers proved to be polymorphic displaying up to 33 alleles per marker (Table 1). Of the 162 isolates that were analyzed, 13 strains yielded multiple peaks as the most likely result from mixed cultures [21] and these were excluded from further analysis. These 13 samples were from allergic fungal rhinosinusitis (n = 8), pulmonary aspergillosis (n = 2), keratitis (1), invasive rhinosinusitis (1), cerebral granuloma (1) showing that in approximately 9 percent of all cases, more than 1 isolate could be involved. For the remaining 149 isolates, the discriminatory power for the individual markers ranged from 0.657 to 0.954 (Table 2). The panel of all markers combined yielded a ‘D’ value of 0.997. Incidentally, no amplification product was obtained for markers 3C, 4A or 4C in <2% of all isolates possibly due to (a) polymorphism(s) underneath the amplification primer(s) or as the result of deletion of the locus from the genome. In ∼15% of all isolates, a deletion of 1 bp was observed in the flanking region of the repeat in marker 3B leading to so-called n.2 alleles (as being the same size as n repeats plus 2 extra bases) [21].

**Table 2:** Discriminatory power (D) of individual markers, sets of markers and the entire panel of markers.

Marker	D	Set	D	Panel	D
AflaSTR2A	0.954	AflaSTR2	0.992	AflaSTR	0.997
AflaSTR2B	0.736				
AflaSTR2C	0.841				
AflaSTR3A	0.657	AflaSTR3	0.992		
AflaSTR3B	0.944				
AflaSTR3C	0.910				
AflaSTR4A	0.675	AflaSTR4	0.950		
AflaSTR4B	0.788				
AflaSTR4C	0.746				

In the collection of 149 pure isolates 124 different genotypes could be recognized. Among all genotypes, 105 genotypes were only found once. Fourteen genotypes were shared between two isolates, four genotypes were shared between three isolates and one genotype was shared by four isolates. Analysis of the details of those four isolates in one genotype revealed that two of them were isolated from patients with post-operative endophthalmitis, who were operated in an eye camp. Two isolates that were isolated form corneal scrapings of patients suffering from keratitis were of the same genotype. Fourteen small clusters were observed of related genotypes that differed in only a single marker from each other. Each of these clusters contained two to five isolates. Within each cluster, the differences between the genotypes were mostly due to variations in markers 2A and 3B which were also the most discriminatory markers in the entire panel.

**Figure 1:**
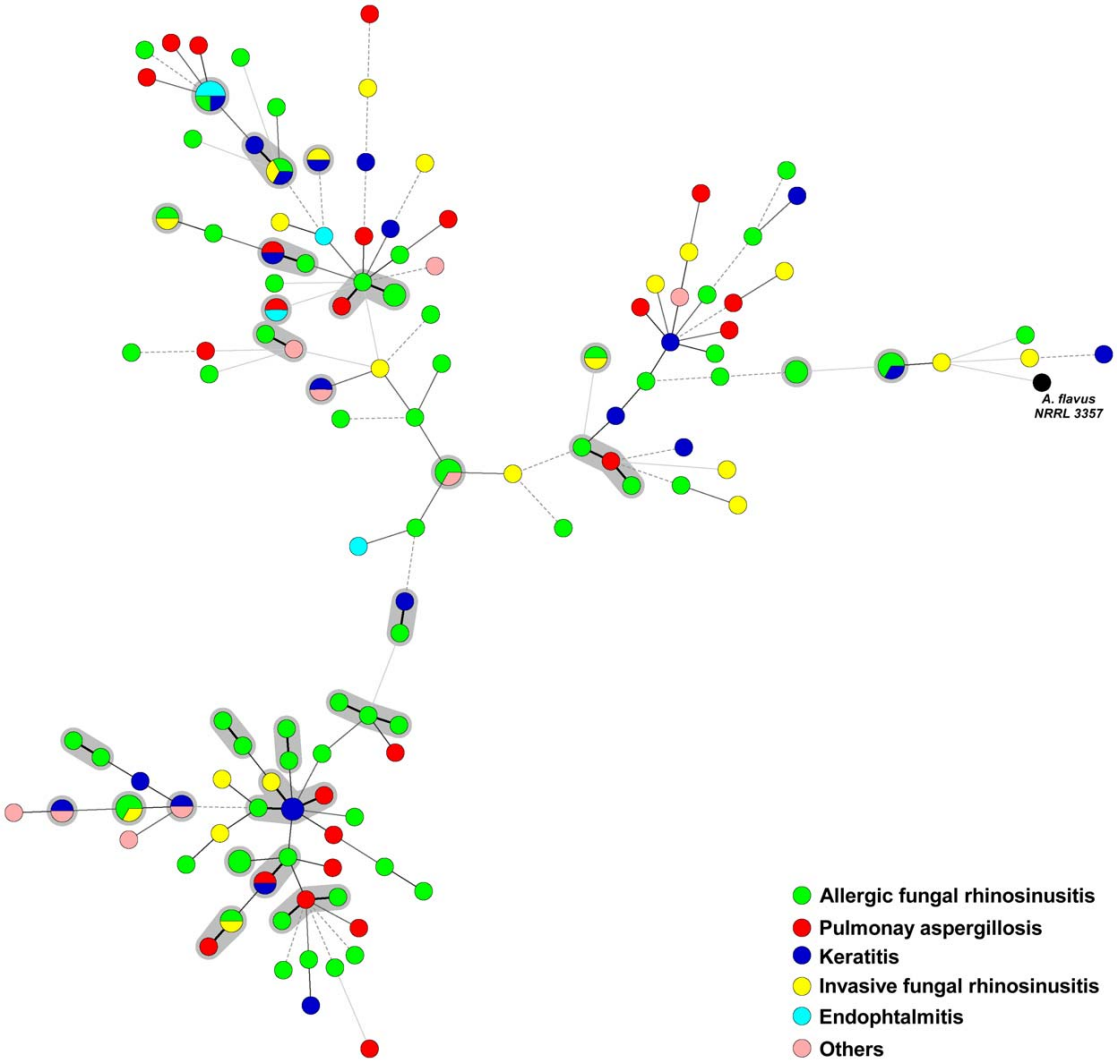
Graphical representation of the results. Minimum spanning tree representing the genotypic diversity of 149 clinical *A. flavus* isolates and a reference isolate using microsatellite typing. The MST is based on a categorical analysis of the data. Each circle represents a unique genotype. The size of the circle corresponds to the number of isolates with the same genotype. The thickness and size of the connecting bars correspond to the number of different markers between linked genotypes. The black genotype is from the reference strain *A. flavus* NRRL 3357. Genotypes with a shaded background contain 2 or more isolates with identical genotypes or contain genotypes that differ in only 1 microsatellite marker as the possible result of microevolutionary events and are likely to be clonally related.

A graphical illustration of the observed genotypes showed no correlation between the genotype of the involved isolate and clinical presentation of the infection (Figure 1).

**Figure 2:**
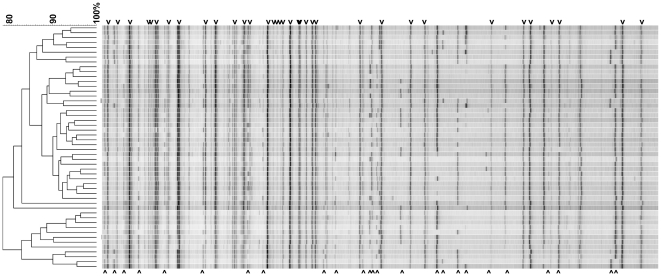
AFLP fingerprints of *A. flavus*. Representative fingerprints are shown for 50 randomly chosen isolates. The dendrogram is based on UPGMA clustering using the Pearson correlation coefficient. The scale bare indicates the percentage similarity. The fingerprints shown are restricted to DNA fragments in the range of 50–300 nt. Indicated with arrowheads above the fingerprints are some of the invariable bands, present in all isolates, that may constitute the core-genomic elements of *A. flavus*. Indicated with arrowheads below the fingerprints are some of the variable bands enabling discrimination between individual isolates.

All isolates were also analyzed by AFLP analysis. Figure 2 shows representative results for 50 randomly chosen isolates. In addition to the well recognizable invariable DNA bands that are present in all isolates tested, also multiple variable DNA bands are visible that are present in some but not all isolates. Based on these variable bands, almost all isolates can be distinguished from each other. Isolates with identical microsatellite genotypes also had indistinguishable AFLP fingerprints. The large number of invariable bands, together with the overall high similarity of 80% or more between the fingerprints, are in agreement with a monophyletic origin of the isolates.

## Discussion

With *A. flavus* being the second most important *Aspergillus* species to cause invasive infection and most common *Aspergillus* species in sinusitis and endophthalmitis in tropical countries [4]–[6], there is a clear need for improved molecular typing methods in order to study the molecular epidemiology of this fungus. The panel of nine markers presented in this study provides excellent discriminatory power for *A. flavus* strains dividing our 149 *A. flavus* strains into 124 genotypes. Microsatellite based typing has emerged as a reproducible and discriminatory typing technique for strain typing of *Aspergillus* species. Several microsatellite panels have already been described for successful discrimination between *A. fumigatus* isolates from various origins [21], [25], [26]. Compared to other molecular typing techniques, use of microsatellites is simple, relatively inexpensive, and highly discriminatory [29], [30]. By employing allelic ladders, the technique should also easily allow exchange of results between laboratories [20]. The availability of the *A. flavus* full genome sequence in the public domain allowed us to search for suitable microsatellite markers *in silico* and to develop the present typing method for *A. flavus*.

When pattern based techniques are employed, it is difficult to recognize if a sample happen to be a mixture of different genotypes. With the use of microsatellites mixed cultures are easily recognized by the presence of multiple peaks for most of the markers. In this study, 13 isolates appeared to be of a mixed culture indicating that people may be colonized and/or infected by more than one *A. flavus* isolate simultaneously and that any isolate from a clinical specimen does not necessarily correspond to the one(s) actually causing disease.

Analysis of the complete genome sequence of *A. flavus* has revealed the presence of functioning mating type locus (MATI-1 and MATI-2) [31], [32]. Recently, Horn et al [33] described the sexual state of this fungus by crossing between strains of the opposite mating types. They also demonstrated the occurrence of genetic recombination in *Petromyces parasiticus*, a fungus very closely related to *Petromyces flavus* (teleomorphic state of *A. flavus*). They hypothesized that recombination during sexual reproduction in *P. flavus* accounts for greater variation in the genome of this fungus [34]. Our findings of the high degree of genetic diversity obtained using both microsatellite and AFLP analyses are in excellent agreement with a sexual mode of reproduction in *A. flavus in-vivo*. Nevertheless, there are also clear signs of clonal expansion since several clusters of isolates were of almost identical genotypes as the likely result of clonal expansion and/or microevolutionary events leading to small repeat numbers changes in the most discriminatory microsatellite markers (Figure 1).

In the present study all the isolates were presumed to be unrelated as they were collected from different patients, from multiple provinces and were collected over a 2 year period. However, several of the observed genotypes were found more than once. One genotype was recognized in four isolates. Two of those isolates were from patients with post-operative endophthalmitis. Those two patients were from same locality and were operated for cataract surgery in the same rural eye camp. This finding suggests that this panel of microsatellite markers would be an interesting alternative to the other reported techniques for local outbreak investigations. Recently, Hadrich et al. reported microsatellite typing of *A. flavus* to trace an infection in hematology unit [35]. They showed that at least in two patients the isolate causing invasive aspergillosis and the environmental isolate belonged to the same genotype indicating hospital acquired colonization or infection. The panel of microsatellite markers in their study had one marker in common with the panel presented here (AflaSTR4B).

The results of the present study indicate that there is no apparent correlation between the genotype of the isolates and the clinical disease. Probably the host factor plays a more important role in the clinical presentation of an infection than the genotype of the involved isolate(s) and any genotype may cause an infection at any site. This appears not be unique for *A. flavus*, since this has also been shown for, for instance, *A. fumigatus* [36], [37].

In conclusion, we describe a highly discriminatory microsatellite panel for discrimination between *A. flavus* isolates of various origins. The multicolor multiplex approach used in this study helped in typing large number of isolates in short time. Furthermore, the advantages of this approach are its high reproducibility, exchangeability of results and possibility of development of databases for future comparison of data. This technique may be used to investigate any outbreak due to *A. flavus*.
